# Medical College Fees

**Published:** 1869-07-15

**Authors:** Wm. K. Bowling, Geo. Bayless

**Affiliations:** President; Library Medical Department University of Louisville; Secretary; Library Medical Department University of Louisville


### *Medical College Fees.*

LIBRARY MEDICAL DEPARTMENT UNIVERSITY OF LOUISVILLE,
May 27, 1869.

At a meeting of Delegates from Medical Colleges for the purpose of considering the question of fees, which was held this day, the following colleges were represented by delegates or letter, viz.:

University of Nashville; Shelby Medical College, Nashville; Memphis Medical College; St.Louis Medical College; Humboldt Medical College of St. Louis; Rush Medical College of Chicago; Chicago Medical College; Indiana Medical College of Indianapolis; Miami Medical College of Cincinnati; University of Louisville.

On motion, Dr. Bowling was elected Chairman, and Dr. Bayless Secretary of the meeting. After a prolonged conference, the following preamble and resolutions were adopted:

WHEREAS, The call for a convention of Delegates from the Medical Colleges of the West, for the purpose of arranging a uniform scale of fees, sent by the Faculty of the Medical Department of the University of Louisville to the Colleges of Nashville, Memphis, Cincinnati, Columbus, Cleveland, Detroit, Chicago, St. Louis, Indianrpolis and Louisville, has met with a cordial response in person or by letter from a majority of said Colleges, and

WHEREAS, The fact that several of said Colleges have issued their announcements for the ensuing sessions makes definite action at present impossible, and

WHEREAS, The views and opinions of the various schools as given by delegates and letters differ greatly; therefore be it

*Resolved*, That it is the hope of this Convention that a universal scale of charges shall be adopted by all the Medical Colleges of our country, and we do most earnestly advise such a scale shall be agreed upon; and it is our belief that the glory and usefulness of our profession would be enhanced by the highest rate advised by the American Medical Association.

*Resolved*, It is not less to be hoped that all the Medical Colleges of our country would fix a higher standard of preliminary and medical education as a pre-requisite for graduation.

*Resolved*, That the Convention request all the Medical Colleges in the United States to send each one delegate to a meeeing to be held in Washington, on Monday, May 2, 1870, to take efficient steps toward carrying out in good faith the recommendations of the American Medical Association in reference to medical education, and also to form a permanent association of American medical teachers.

*Resolved*, That a copy of these proceedings be sent to all the medical journals in the country. WM. K. BOWLING, *President*.

GEO. BAYLESS, *Secretary*.

